# Non-encapsidated miRNA contaminants found in AAV preparations

**DOI:** 10.1016/j.omtm.2024.101336

**Published:** 2024-09-20

**Authors:** Mark A. Brimble, Stephen M. Winston

**Affiliations:** 1 Department of Host-Microbe Interactions, St. Jude Children’s Research Hospital, Memphis, TN 38105, USA; 2 Department of Surgery, St. Jude Children’s Research Hospital, Memphis, TN 38105, USA; 3 Graduate School of Biomedical Sciences, St. Jude Children’s Research Hospital, Memphis, TN 38105, USA

## Main text

Adeno-associated virus (AAV) has been in clinical use for gene therapy applications for over a decade, with 6 FDA-approved drug products at the time of writing.^1^ However, this vector is manufactured in a complex biological system, and it is currently an elusive possibility to produce an AAV gene therapy product devoid of nucleic acid contaminants. In recent years, there has been extensive work profiling and understanding the unintended DNA molecules contained within preparations of AAV.^2^ This has increased awareness of the causes of these DNA impurities and innovation to minimize their encapsidation within AAV particles. In a new article, Penaud-Budloo and colleagues examine a previously unexplored consideration: the RNA content of recombinant AAV (rAAV) preparations.^3^

In-depth analysis of vector purity has typically used next-generation sequencing (NGS) without a reverse transcription step, precluding the identification of any RNA present in the prep.^4^^,^^5^^,^^6^ In this study, by intentionally examining AAV preparations for RNA content within, the authors immediately observed the presence of RNA molecules. They found a bias toward smaller RNA species in the purified AAV preparations compared to the RNA composition of the production cell lines. Specifically, due to their inherent stability, the group then focused on assaying the presence of microRNA (miRNA) within purified AAVs, finding that the most abundant miRNAs in purified AAVs were the most abundant in the production cells. miRNAs were detected across the serotypes tested and found in vector preparations produced in both major cell culture platforms for rAAV manufacturing: HEK293 and SF9 cells.

The obvious question arising is are these detectable miRNAs a mere result of carry through during purification or are they encapsidated? Fortunately, it appears that the vast majority of these miRNAs are external to the vector particles. The concentration of vector with a 100 kDa Amicon filter and digestion with RNA-specific nucleases reduced the miRNA presence within the preparations by orders of magnitude. As one would expect for non-encapsidated sequences, the levels of miRNAs in the final product were also influenced by the purification technique. There was a greater abundance of miRNAs in rAAVs purified using cesium chloride (CsCl) gradient ultracentrifugation as compared to immunoaffinity chromatography. The highest level of miRNAs was found in the empty capsid fraction of the CsCl-purified vector. Interestingly, the final figure suggests that a small portion of the detected miRNAs may be packaged within the viral particles. A miRNA sequence mimic (miR-19b mimic) was spiked into an AAV preparation prior to treatment with RNase A or a combination of RNase A and RNase T1. There was a significant reduction in detectable sequences for both miRNAs. However, the degree of enzymatic degradation was greater with the spiked-in miR-19b mimic as compared to the contaminating miR-184 originating from the production cell line. While this strongly suggests that some of the contaminating miRNA is packaged into AAV capsids, further work is needed to say conclusively that a portion of the miRNAs derived from the production cell line are encapsidated within rAAV particles.

It is certainly promising that these miRNA molecules are, at least largely, non-encapsidated. Theoretically, this limits their transferability to cells post-dosing and makes their removal from clinical batches an easier task. Though it should be noted that extra-viral DNA in AAV preps can be immunostimulatory,^7^ and so these miRNAs should not be dismissed out of hand simply because they are not found within the AAV particles themselves. Moreover, while the group focused on the presence of miRNAs, their electropherogram data suggest that longer RNA molecules can also be detected. It will be important to assay whether these longer RNAs are also a result of purification carry-through or whether any RNA is capable of being packaged into AAV particles during rAAV production.

For clinical AAV production, good manufacturing practice (GMP) purification protocols likely include steps that would render the removal of these miRNAs, and any other non-encapsidated RNA, during downstream processing. Indeed, this is indicated by the chromatographic purification and 100 kDa Amicon filtration steps in one previously published GMP protocol for AAV8 production.^8^ Perhaps this means there is little cause for concern in clinical-grade preparations. However, AAV purification protocols vary between organizations and drug products, so this assumption cannot be guaranteed. For instance, Benzonase is a popular nuclease for AAV purification. However, it has been previously shown that Benzonase does not affect the degradation of miRNA and that RNase A is a far more effective agent for this task.^9^ These observations were recapitulated in this study: the purified AAV was treated with Benzonase during the initial purification procedures before analysis of the vector preps for RNA content.

Now that it has been demonstrated that RNA can be present within purified AAV preparations, what does this mean for the characterization of clinical-grade material? The non-binding FDA recommendations for gene therapy products set a guideline of less than 10 ng of residual DNA <200 bp within clinical products.^10^ These guidelines currently do not reference residual RNA levels. While it is encouraging from a safety standpoint that these contaminants appear to be outside of the vector particles, the ready detection of miRNA in purified AAV within this study strongly suggests that, at a minimum, measurement of RNA content of the final vector preparation should be undertaken and reported on for products destined for clinical use.

In conclusion, the study from Penaud-Budloo and colleagues reveals a new consideration for purity testing rAAV preparations and an avenue of further work to assess other RNA species found within purified AAV. Gene therapy for genetically driven pathologies is already realizing a transformational potential in the lives of treated patients. As gene therapy becomes more routine and widespread, it is incumbent on the field to ensure that these novel medicines contain the minimum impurities feasible with the current iterations of the technology. This manuscript serves as an important reference point for those involved in clinical rAAV gene therapy manufacturing and indicates that an assessment of RNA content should be included in vector purity considerations.

## Acknowledgments

M.A.B. is supported by funding from the 10.13039/100000897Cystic Fibrosis Foundation (005027F5222).

## Declaration of interests

M.A.B. is a listed inventor on pending (WO2021242664A1) and provisional patents related to the reduction of DNA impurities in rAAV preparations. S.M.W. is a listed inventor on provisional patents related to the reduction of DNA impurities in rAAV preparations.
